# Molecular analysis of methanogenic archaea in the forestomach of the alpaca (*Vicugna pacos*)

**DOI:** 10.1186/1471-2180-12-1

**Published:** 2012-01-05

**Authors:** Benoit St-Pierre, André-Denis G Wright

**Affiliations:** 1 Department of Animal Science, The University of Vermont, 570 Main Street, Burlington, VT 05405, USA; 2 Department of Medicine, The University of Vermont, 111 Colchester Ave., Burlington, VT 05401, USA; 3 Department of Microbiology and Molecular Genetics, The University of Vermont, 95 Carrigan Drive, Burlington, VT 05405, USA

## Abstract

**Background:**

Methanogens that populate the gastrointestinal tract of livestock ruminants contribute significantly to methane emissions from the agriculture industry. There is a great need to analyze archaeal microbiomes from a broad range of host species in order to establish causal relationships between the structure of methanogen communities and their potential for methane emission. In this report, we present an investigation of methanogenic archaeal populations in the foregut of alpacas.

**Results:**

We constructed individual 16S rRNA gene clone libraries from five sampled animals and recovered a total of 947 sequences which were assigned to 51 species-level OTUs. Individuals were found to each have between 21 and 27 OTUs, of which two to six OTUs were unique. As reported in other host species, *Methanobrevibacter *was the dominant genus in the alpaca, representing 88.3% of clones. However, the alpaca archaeal microbiome was different from other reported host species, as clones showing species-level identity to *Methanobrevibacter millerae *were the most abundant.

**Conclusion:**

From our analysis, we propose a model to describe the population structure of *Methanobrevibacter*-related methanogens in the alpaca and in previously reported host species, which may contribute in unraveling the complexity of symbiotic archaeal communities in herbivores.

## Background

Enteric methane emitted by livestock species is produced by symbiotic methanogens which use as substrates the CO_2 _and H_2 _that result from digestion of plant fibers in the gastrointestinal tract of their host. Because it is not assimilated, methane is released into the environment, mostly through eructation [1]. Since this process results in a loss of energy from the host [2], reducing methane emissions would then not only be beneficial for climate control, but also for enhancing livestock efficiency and productivity. To achieve these goals, an essential first step is the identification of rumen methanogens and characterization of their phylogeny. A number of studies using culture-independent methods such as 16S rRNA gene identification have revealed that a great diversity of methanogens populate the rumen, which vary depending on factors such as host species and diet [3].

It has also become apparent that the analysis of methanogen populations in traditional livestock species would greatly benefit from investigating methanogen communities in other herbivores [4-6]. Camelids represent an interesting group because they are evolutionarily distant from ruminants. They originated in North America approximately 40-45 million years ago (mya), where they diversified and remained confined until 3.5-6 mya, when representatives arrived in Asia and in South America [7]. The natural geographical distribution of modern camelid species reflects this ancestral separation: the Dromedary resides in northern Africa and south-west Asia, the Bactrian camel is found in central Asia, whereas the llama and alpaca are located in South America.

Alpaca populations are rapidly growing world-wide, because of the fine texture and quality of the wool fiber produced by this species. This economic pursuit has in turn sparked interest in its biology, revealing that the alpaca is an adaptive feeder, ranging from grasses and hay to shrubs and trees, that requires less energy and protein input for growth and maintenance than domesticated ruminants [8,9]. In contrast to the four-chambered stomach of ruminants, camelids such as the alpaca possess a three-chambered stomach whose physiology has been actively investigated to determine its contribution to the higher production efficiency of these animals [10-16].

Because the alpaca is also very efficient at digesting plant cell wall material and produces less methane [8,14], its gastrointestinal microbial community also likely contributes significantly to its digestive efficiency. In contrast to ruminants, gut microbiomes remain largely uncharacterized in alpacas, with limited reports on the diversity and density of protozoa [17,18] or bacterial populations [19], and no published studies on methanogenic archaea populations. In this context, the increased efficiency of the alpaca combined with its low methane production makes it a very attractive host model to study methanogens. Based on the anatomy and physiology of the alpaca digestive system, we hypothesized that the composition and structure of its microbial populations may be different than in previously reported ruminant species. To test our hypothesis, we investigated the composition of methanogen populations in the forestomach of five alpacas by sequencing and analyzing the molecular diversity of methanogen 16S rRNA genes from individually constructed clone libraries. The specific objectives of our study were to identify methanogens that reside in the foregut of alpacas and to determine their phylogeny.

## Methods

### Animal sampling

All procedures were approved under The University of Vermont's Institutional Animal Care and Use Committee (IACUC) protocol 11-021, and Institutional Biosafety Committee (IBC) protocol 10-029. Five male alpacas, fed a mixture of timothy, clover and rye supplemented with fresh fruits (bananas and apples), and maintained under normal conditions at the Hespe Garden Ranch and Rescue (http://www.hespegarden.com/, Washington, Vermont, USA), were stomach tubed while sedated by a licensed veterinarian. Forestomach samples (20 ml), which included partially digested feed and fluid, were kept on ice and then frozen at –20°C on the day of collection. Samples were maintained frozen until DNA extraction. Age at sampling was 19 months (alpaca 9), 21 months (alpaca 6), 32 months (alpacas 5 and 8) and 7.5 years (alpaca 4).

### Microbial DNA isolation, clone library construction, sequencing and real-time PCR

Microbial DNA from forestomach samples was isolated as described by Yu and Morrison [20]. Methanogen 16S rRNA genomic sequences were amplified from purified forestomach microbial DNA by PCR using the methanogen-specific primers Met86F and Met1340R [21]. PCR reactions were performed with Taq polymerase from Invitrogen (USA) on a C1000 Thermal Cycler (BioRad) under the following conditions: hot start (4 min, 95°C), followed by 35 cycles of denaturation (30s, 95°C), annealing (30s, 58°C) and extension (2 min, 72°C), and ending with a final extension period (10 min, 72°C). Methanogen 16S rRNA gene libraries were constructed by cloning PCR-amplified products from each forestomach DNA sample into the pCR2.1-TOPO vector, using the TOPO TA cloning kit (Invitrogen, USA). Recombinant plasmids from bacterial clones negative for α-complementation in the presence of X-gal (5-bromo-4-chloro-3-indolyl-beta-D-galactopyranoside) were screened by colony-PCR with the M13 Forward and M13 Reverse primers. PCR products from positive bacterial clones were used directly as templates for Sanger DNA sequencing with the new forward and reverse primers Met643F (5'-GGA CCA CCW RTG GCG AAG GC-3') and Met834R (5'-CTT GCG RCC GTA CTT CCC AGG-3'). Nucleotide sequencing was performed by the DNA Analysis Facility at the Vermont Cancer Center (The University of Vermont). Real-time PCR was used to estimate cell densities from forestomach contents of individual alpacas using the mcrA-F and mcrA-R primer pair as described by Denman et al. [22].

### Computational analysis of nucleotide sequences

ChromasPro (Version 1.5, Technelysium Pty Ltd) was used to proofread the methanogen 16S rRNA gene sequences from positive clones and assemble them into contigs of 1 255-1 265 bp in length. Each clone was designated by "AP" to indicate it originated from alpaca, the animal sampled (4, 5, 6, 8 or 9) and a specific identification number.

Library clones were grouped into operational taxonomic units (OTU), based on a 98% sequence identity cutoff [6], by the open-source program MOTHUR [23], which used distance data generated from the combined clone libraries by the Kimura two-parameter model [24] in PHYLIP (Version 3.69 [25]). MOTHUR was also used to generate a rarefaction curve, determine the Chao1 richness estimator, and calculate the Shannon and LIBSHUFF diversity indices. OTU coverage (C) was calculated using the equation C = 1-(n/N) × 100, where n is the number of OTUs represented by a single clone and N is the total number of clones analyzed in the library. Identification of representative OTU sequences was performed using the BLAST search engine http://blast.ncbi.nlm.nih.gov/Blast.cgi against the NCBI nucleotide sequence database [26].

For phylogenetic reconstruction, 51 alpaca methanogen 16S rRNA sequences (one representative from each alpaca OTU) were combined with 45 methanogen 16S rRNA gene sequences representing major archaeal phylogenetic groups. PHYLIP (Version 3.69 [25]) was used to construct a neighbor-joining tree [27], which was bootstrap resampled 1,000 times.

### Nucleotide sequence accession numbers

The sequences from this study have been deposited in the GenBank database under the accession numbers JF301970-JF302647. For a detailed list of clones and accessions, see Additional file 1: Table S1.

## Results

### Phylogenetic analysis of methanogenic archaea in the alpaca forestomach

**Figure 1:**
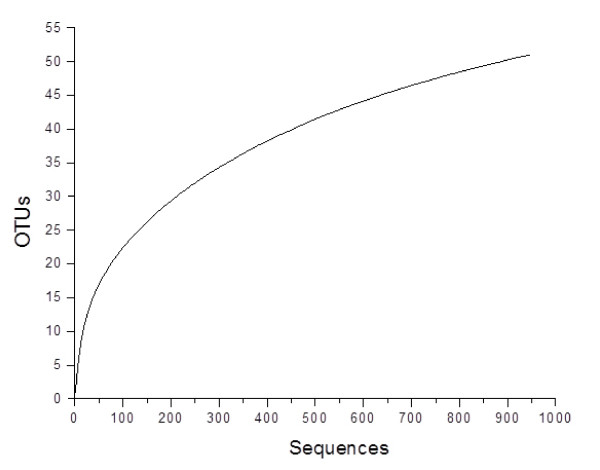
**Collector's rarefaction curve of observed species-level OTUs generated by MOTHUR **[23]** using a 98% identity cutoff value**.

We investigated the diversity and phylogeny of methanogenic archaea in the forestomach of the alpaca by constructing individual methanogen 16S rRNA gene clone libraries from five animals. The number of non-chimeric clones isolated per individual library ranged from 179 to 201, for a combined total of 947 methanogen 16S rRNA gene sequences for analysis in our study. Based on a 98% sequence identity criterion, established from the level of identity that exists between 16S rRNA genes from validly characterized *Methanobrevibacter *species [6], our combined library sequences were grouped into 51 distinct OTUs (Table 1). Clones were unevenly distributed between OTUs, with 80.8% of sequences grouped within OTUs 1-10, compared with 19.2% for the remaining 41 OTUs. We used 2 different methods to assess the depth of coverage and sampling efficiency of our study at the OTU level. While the calculated rarefaction curve proved to be non-asymptotic, it approached the saturation point (Figure 1), which we conservatively estimated to be 63 OTUs using the Chao1 richness indicator. Coverage (C) for individual and combined libraries was greater than 90% at the OTU level (Table 2). Together, these results support that the sampling efficiency of our study was very high.

**Table 1:** OTU distribution of clones between individual alpaca animals

OTU	Nearest Valid Taxa	% Seq. Identity	Alpaca 4	Alpaca 5	Alpaca 6	Alpaca 8	Alpaca 9	Total Clones
**1**	*Mbr. ruminantium*	98.8	29	22	13	54	21	139
**2**	*Mbr. millerae*	98.1	27	15	49	12	7	110
**3**	*Mbr. millerae*	98.3	20	35	26	19	9	109
**4**	*Mbr. millerae*	99.0	33	1	16	4	55	109
**5**	*Mbr. millerae*	98.5	16	13	21	17	15	82
**6**	*Mbr. gottschalkii*	97.8	2	35	13	5	2	57
**7**	*Mba. alcaliphilum*	96.8	8	21	6	7	13	55
**8**	*Mbr. millerae*	97.9	2	0	4	31	5	42
**9**	*Mbr. millerae*	97.9	10	4	4	11	2	31
**10**	*Mbr. millerae*	99.8	4	1	12	0	14	31
**11**	*Mbr. smithii*	98.1	5	9	3	4	4	25
**12**	*Mbr. millerae*	97.9	0	19	3	0	2	24
**13**	*Msp. stadtmanae*	96.4	3	3	0	2	9	17
**14**	*Mbr. smithii*	98.0	6	1	4	5	0	16
**15**	*Apr. boonei*	82.3	0	0	9	0	0	9
**16**	*Mbr. ruminantium*	96.4	3	2	1	0	1	7
**17**	*Mbr. millerae*	98.7	3	0	2	0	2	7
**18**	*Apr. boonei*	82.5	0	0	1	1	4	6
**19**	*Mba. alcaliphilum*	95.5	1	1	2	1	0	5
**20**	*Mba. alcaliphilum*	96.5	0	4	1	0	0	5
**21**	*Mbr. olleyae*	96.7	0	1	0	3	1	5
**22**	*Msp. stadtmanae*	96.5	0	0	1	4	0	5
**23**	*Mbr. millerae*	97.2	1	0	1	2	0	4
**24**	*Mba. alcaliphilum*	96.9	1	0	0	0	3	4
**25**	*Mbr. ruminantium*	98.4	0	1	1	0	1	3
**26**	*Mbr. ruminantium*	97.7	0	2	1	0	0	3
**27**	*Mbr. smithii*	97.3	0	1	1	1	0	3
**28**	*Apr. boonei*	82.6	0	0	2	1	0	3
**29**	*Mbr. millerae*	97.3	2	0	0	0	0	2
**30**	*Mbr. millerae*	97.8	2	0	0	0	0	2
**31**	*Apr. boonei*	81.6	0	2	0	0	0	2
**32**	*Mbr. ruminantium*	97.5	0	1	0	1	0	2
**33**	*Mbr. ruminantium*	97.2	0	1	0	0	1	2
**34**	*Mbr. ruminantium*	95.6	0	0	1	1	0	2
**35**	*Apr. boonei*	81.7	0	0	1	0	1	2
**36**	*Mbr. gottschalkii*	96.4	0	0	0	0	2	2
**37**	*Mbr. gottschalkii*	96.7	1	0	0	0	0	1
**38**	*Apr. boonei*	80.9	0	1	0	0	0	1
**39**	*Mbr. ruminantium*	96.4	0	1	0	0	0	1
**40**	*Mbr. ruminantium*	94.8	0	1	0	0	0	1
**41**	*Mbr. wolinii*	95.8	0	1	0	0	0	1
**42**	*Mbr. millerae*	97.2	0	0	1	0	0	1
**43**	*Mbr. ruminantium*	96.8	0	0	1	0	0	1
**44**	*Mbr. olleyae*	96.7	0	0	0	1	0	1
**45**	*Mbr. smithii*	97.5	0	0	0	1	0	1
**46**	*Mbr. millerae*	96.2	0	0	0	1	0	1
**47**	*Msp. stadtmanae*	95.7	0	0	0	0	1	1
**48**	*Apr. boonei*	81.7	0	0	0	0	1	1
**49**	*Mbr. millerae*	96.1	0	0	0	0	1	1
**50**	*Mbr. millerae*	97.3	0	0	0	0	1	1
**51**	*Mbr. millerae*	95.4	0	0	0	0	1	1
**Total**			179	199	201	189	179	947

*Apr*. = *Aciduliprofundum*; *Mba*. = *Methanobacterium*; *Mbr*. = *Methanobrevibacter*; *Msp*. = *Methanosphaera*.

**Table 2:** Coverage, Shannon Index, and LIBSHUFF method calculated using MOTHUR^a ^for each methanogen 16S rRNA gene clone library

Clone Library	No. of unique OTUs	% OTU coverage	Shannon Index ± 95% confidence limits	**LIBSHUFF Method**^**c**^
Alpaca 4	3	97.8	2.06 ± 0.15^b^	*P*≤ 0.0004
Alpaca 5	5	93.5	2.12 ± 0.14^b^	*P*≤ 0.0022
Alpaca 6	2	94.0	1.96 ± 0.15^b^	*P*≤ 0.0001
Alpaca 8	3	95.2	1.89 ± 0.16^b^	*P*≤ 0.0028
Alpaca 9	6	94.4	2.09 ± 0.17^b^	*P*≤ 0.0028
**Combined**	-	98.4	2.85 ± 0.07^b^	-

^a ^Schloss et al. [23]

^b ^No significant difference between these values

^c ^LIBSHUFF Method calculated for each pair of methanogen 16S rRNA gene clone libraries (e.g. the comparison of Alpaca 4 against Alpaca 5, Alpaca 6, Alpaca 8, and Alpaca 9 was always significantly different *P *≤ 0.0004)

We found that 37 OTUs, representing 88.3% of clones isolated from our combined libraries, displayed 95% or greater genus-level sequence identity to species belonging to *Methanobrevibacter*, making it the dominant genus in the microbial community of the alpaca forestomach (Table 3). Within this category, six OTUs (2, 3, 4, 5, 10 and 17), accounting for 47.3% (448/947) of all clones, had 98% or greater species-level sequence identity to *Methanobrevibacter millerae *(Table 3). In contrast, only 15% (142/947) of library clones that were grouped into two OTUs (1 and 25) showed species-level sequence identity to *Methanobrevibacter ruminantium*, and only 4.3% (41/947) of clones populating two OTUs (11 and 14) displayed over 98% sequence identity to *Methanobrevibacter smithii*. Clones from 27 OTUs (21.1% or 200/947 of sequences from the combined libraries) only had 95-97.9% sequence identity to validly described *Methanobrevibacter *species (Tables 1 and 3), and likely corresponded to methanogen species that have yet to be cultivated. Based on 16S rRNA sequence identity, there is likely to be overlap between different hosts in representation of these uncharacterized methanogens, such as for instance AP5-146 (OTU 41) which was almost identical (1265/1268 bp) to the Ven09 methanogen clone identified in sheep from Venezuela [28].

**Table 3:** Percentage (%) in 16S rRNA gene clone distribution by taxon or phylum between alpacas

Taxa	Alpaca 4	Alpaca 5	Alpaca 6	Alpaca 8	Alpaca 9	Combined
***Methanobacteriales***						
***Methanobrevibacter***						
***ruminantium ***^1^	16.2	11.6	7.0	28.6	12.3	15.0
***millerae ***^1^	57.5	32.7	62.7	27.5	57.0	47.3
***smithii ***^1^	6.1	5.0	3.5	4.8	2.2	4.3
**unassigned **^2^	12.8	34.7	15.4	30.7	10.6	21.1
***Methanobacterium***						
**unassigned **^2^	5.6	13.1	4.5	4.2	8.9	7.3
***Methanosphaera***						
**unassigned **^2^	1.7	1.5	0.5	3.2	5.6	2.4
***Thermoplasmatales***						
**unassigned **^3^	0.0	1.5	6.5	1.0	3.4	2.5

^1^sequences in OTUs that have 98% or higher sequence identity to the 16S rRNA gene of the specified species

^2^sequences in OTUs that have 95-97.9% sequence identity to the16S rRNA gene of a valid species from the specified genera

^3^sequences in OTUs that have 80-83% sequence identity to the 16S rRNA gene of *Aciduliprofundum boonei *and are likely part of a new order of uncultured archaea

**Figure 2:**
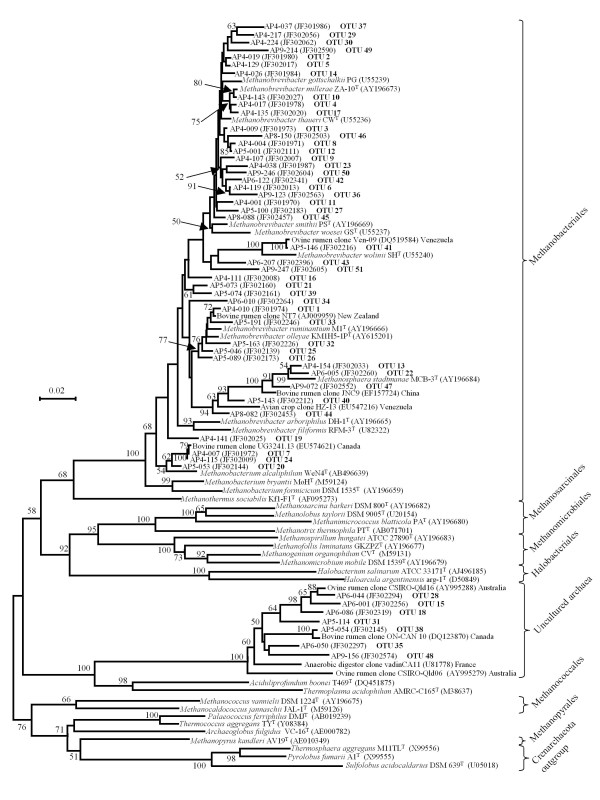
**A neighbor-joining distance matrix tree of the archaea in the alpaca forestomach derived from 16S rRNA gene evolutionary distances produced by the Kimura two-parameter correction model **[24]. Bootstrap supports are indicated as a percentage at the base of each bifurcation. Bootstrap values less than 50% are not shown. Evolutionary distance is represented by the horizontal component separating the species in the figure. The scale bar corresponds to 2 changes per 100 positions.

The remaining 14 OTUs were divided into three distinct phylogenetic groups. Clones from four OTUs (7, 19, 20 and 24), accounting for 7.3% (69/947) of the library sequences, showed 95-97.9% sequence identity to species belonging to the genus *Methanobacterium *(Table 3), and were accordingly grouped in the same cluster (Figure 2). Of interest in this category, clone AP4-007 from OTU 7 was almost identical (1259/1260 bp) to environmental clone UG3241.13 identified in dairy cattle from Canada [29]. Three other OTUs (13, 22 and 47), representing 2.4% (23/947) of clones, displayed genus-level sequence identity to *Methanosphaera *species and were also grouped into a single well-defined cluster by phylogenetic analysis (Figure 2). Finally, 2.5% (24/947) of alpaca clones were phylogenetically very distant from the previously mentioned genera within the order Methanobacteriales (Figure 2), and were grouped into 7 OTUs (15, 18, 28, 31, 35, 38 and 48) (Table 1). While the highest level of sequence identity to a valid methanogen species was 80-83% for all clones in these OTUs (Table 1), these sequences are from a group of methanogens that have consistently been identified in various microbial communities, but they have yet to be validly characterized. In our study, four of the six clones in OTU 18 were 100% identical to CSIRO-Qld19, a 16S rRNA gene sequence identified in the ovine rumen from Australia [30], and the single clone from OTU 38 was identical to ON-CAN.02, a 16S rRNA sequence identified in the bovine rumen from Canada [31]. Of the remaining alpaca sequences in this uncultured group, 16 of 24 clones had 98% or greater sequence identity to previously reported methanogen 16S rRNA genes isolated from rumen samples (data not shown).

### Analysis of methanogen population structure in individual alpacas

In the alpaca 4 library, 16S rRNA gene sequences were distributed between 21 of the 51 combined OTUs, with OTUs 1-5 representing 69.8% (125/179) of clones isolated from this individual (Table 1). We found that 57.5% (103/179) of sequences from alpaca 4 were grouped in OTUs showing 98% or greater sequence identity to *Methanobrevibacter millerae*, while only 12.8% (23/179) were in OTUs that were categorized as unassigned *Methanobrevibacter *sequences (Table 3). Distinctively, alpaca 4 was the only individual for which we did not isolate any clones from the uncharacterized archaeal group (OTUs 15, 18, 28, 31, 35, 38 and 48).

In the alpaca 5 library, sequences were distributed between 27 OTUs, with OTUs 1, 3, 6, 7 and 12 representing the most clones obtained from this individual (66.3%, 132/199). Of note, 16S rRNA gene sequences from alpaca 5 showed the highest representation of unassigned *Methanobrevibacter *OTUs at 34.7% (69/199), as well as the highest representation in unassigned *Methanobacterium *OTUs at 13.1% (26/199) (Table 3). In addition, clones from this individual with species-level identity to *Methanobrevibacter millerae *were relatively under-represented at 32.7% (65/199) compared with alpacas 4, 6 and 9.

In the alpaca 6 library, clones were found in 29 of 51 OTUs, the most within our sampled individuals, with 62.2% (125/201) divided among OTUs 1-5. Remarkably, 62.7% (126/201) of alpaca 6 sequences had species-level identity to *Methanobrevibacter millerae*, the highest representation from any individual, while only 7% (14/201) of its sequences had species-level identity to *Methanobrevibacter ruminantium*, the lowest representation in our study. In addition, sequences from this individual had the highest representation for the uncharacterized archaeal group at 6.5% (13/201), but the lowest representation in unassigned *Methanosphaera *OTUs at 0.5% (1/201) (Table 3).

In the alpaca 8 library, 16S rRNA gene sequences were distributed across 24 of the 51 OTUs, with four OTUs (1, 3, 5 and 8) representing the most clones (64.0%, 121/189) obtained from this individual. Alpaca 8 showed the highest representation (28.6%, 54/189) in OTUs with species-like identity to *Methanobrevibacter ruminantium*, but the lowest representation at 27.5% (52/189) in OTUs having 98% identity or greater to *Methanobrevibacter millerae *(Table 3). In addition, alpaca 8 had a high representation of unassigned *Methanobrevibacter *OTUs with 30.7% (58/189), and a relatively high representation in unassigned *Methanosphaera *OTUs with 3.2% (6/189).

Finally, 16S rRNA gene sequences from the alpaca 9 library were grouped in 27 of 51 OTUs. In this individual, OTUs 1, 4, 5, 7 and 10 represented the most sequences (65.9%, 118/179). Distinctive features of methanogen distribution from this individual were the highest representation in *Methanosphaera*-like OTUs at 5.6% (10/179) and the lowest representation in *Methanobrevibacter*-like OTUs at 10.6% (19/179). The alpaca 9 library also showed a high representation in OTUs with species-like identity to *Methanobrevibacter millerae *(57%, 102/179) and to *Methanobacterium*-like OTUs at 8.9% (16/179).

While individual libraries were found to statistically display similar levels of OTU diversity according to Shannon index comparisons (Table 2), LIBSHUFF analysis indicated that all five individual alpaca libraries were distinct from each other (Table 2) [32]. Density of methanogens in the alpacas sampled in our study ranged between 4.40 × 10^8 ^and 1.52 × 10^9 ^(standard error of the mean: ± 2.02 10^8^) cells per g of forestomach content, as estimated by real-time PCR.

## Discussion

All herbivores rely on mutualistic gastrointestinal microbial communities to digest plant biomass. This process also generates by-products such as methane that are not used by the host and are released into the environment. Methane production by domesticated herbivores is cause for great concern because of its very potent greenhouse gas effect and its negative impact on production as hosts are required to spend energy in order to release methane [33]. Because camelids such as the alpaca exhibit very important differences with ruminants in their dietary preference, the anatomy of their digestive system, their higher feed efficiency, and their lower methane emissions [9], we hypothesized that their digestive system may be populated by distinct methanogens. Using 16S rRNA gene clone libraries constructed from five individual animals, we found that *Methanobrevibacter *phylotypes were the dominant archaea in the forestomach of the alpaca, as it has been reported to be the case in other host species analyzed (for a recent review, please see Kim et al. [3]). Individuals were found to each have between 21 and 27 OTUs, of which two to six OTUs were unique. Although LIBSHUFF analysis indicated that individual clone libraries were significantly different from each other, additional studies comparing a larger pool of animals of different age groups under a controlled diet will be required to gain further insight into individual variation in methanogen population structure in the alpaca. Future studies will also help in assessing the degree to which the methanogen population structure observed in the present study was influenced by factors such as sampling method or a diet not representative of the natural environment of the alpaca.

Methanogen density estimates from our study (4.40 × 10^8 ^- 1.52 × 10^9 ^cells/g) compared favorably with previously reported studies in cattle (9.8 × 10^8 ^cells/g [4] and 1.3 × 10^9 ^cells/g [22]), reindeer (3.17 × 10^9 ^cells/g, [5]), or hoatzin (5.8 × 10^9 ^cells/g [6]). Reduced methane emissions in the alpaca are therefore less likely to be a result of lower methanogen densities, as observed in the wallaby [4], and may be due to differences in the structure of its archaeal community.

Alpaca methanogen populations from our study were distinct in that the most highly represented OTUs showed 98% or greater sequence identity to the 16S rRNA gene of *Methanobrevibacter millerae*. In comparison with other hosts, 16S rRNA clones showing species-like identity to *Methanobrevibacter gottschalkii *were dominant in sheep from Venezuela [28] and in wallabies sampled during the Australian spring time (November sample) [4], but we did not identify any clones from our libraries with species-level sequence identity to this methanogen. In the Murrah breed of water buffalo from India, the majority of clones were from the genus *Methanomicrobium *[34], but we did not detect any 16S rRNA gene sequences from any genera within the order Methanomicrobiales in our analysis. In yak, archaeal sequences related to the *Methanobrevibacter *strain NT7 were the most highly represented [35]. Clones belonging to the uncultured archaeal group were dominant in sheep from Queensland (Australia) [30], wallabies (May sample) [4], reindeer [5], and in potato-fed cattle from Prince Edward Island (Canada) [31], but we found them to be in low abundance in our study. While significantly represented in our libraries, OTUs showing species-level identity to *Methanobrevibacter ruminantium *were not as abundant as reported in the hoatzin [6], in corn-fed cattle from Ontario (Canada) [31], in lactating dairy cattle [36], or in beef cattle fed a low-energy diet [37].

**Figure 3:**
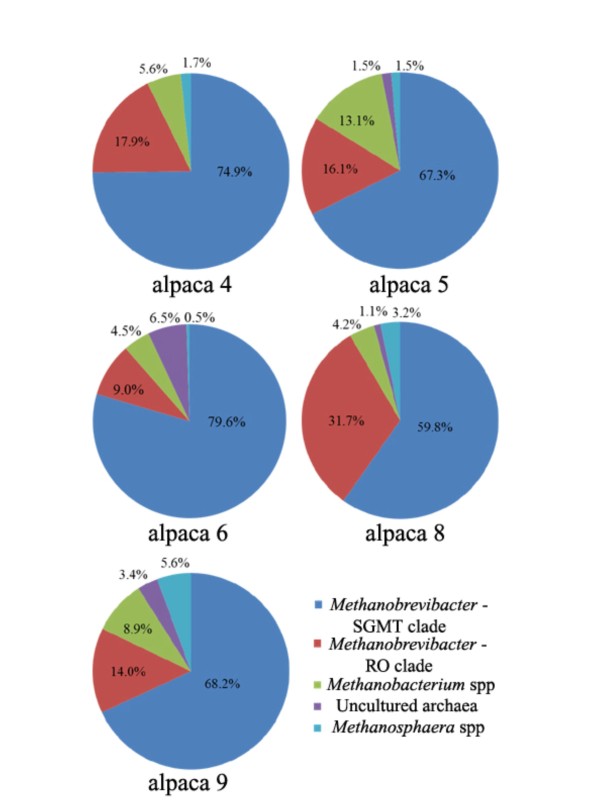
**Pie-chart representation of methanogen 16S rRNA gene clone distribution in each alpaca**. *Methanobrevibacter *sequences that phylogenetically group within the major clade consisting of *Methanobrevibacter smithii, Methanobrevibacter gottschalkii, Methanobrevibacter millerae *and *Methanobrevibacter thaurei *are represented in the *smithii*-*gottschalkii*-*millerae*-*thaurei *clade or SGMT clade. Similarly, the *ruminantium*-*olleyae *or RO clade consists of sequences that phylogenetically group within the major clade consisting of *Methanobrevibacter ruminantium *and *Methanobrevibacter olleyae*.

While their microbiome displayed a distinct representation of specific archaeal groups, alpacas from our study harbored methanogens from similar phylogenetic groups that appeared to form a continuum of species rather than discreet groups (Figure 2), as reported in other hosts [38]. The 37 OTUs from alpaca with genus-like sequence identity to *Methanobrevibacter *species appeared to be mostly distributed between two large clades (Figure 2). One clade consisted of sequences that are closely related to *Methanobrevibacter smithii, Methanobrevibacter gottschalkii, Methanobrevibacter millerae *or *Methanobrevibacter thaurei*, which we referred to as the *smithii*—*gottschalkii*—*millerae*—*thaurei *clade, or simply as the SGMT clade. The other major clade grouped *Methanobrevibacter ruminantium *and *Methanobrevibacter olleyae*—like sequences, which we referred to as the *ruminantium—olleyae *or RO clade. In individual alpaca libraries, the combined representation of sequences from the SGMT and RO clades showed little variation, ranging from 83.4% to 92.8%. However, there were more fluctuations in the representation of the SGMT clade sequences compared to the RO clade between individuals, where clade representation appeared to have an inverse relationship. For instance, in the alpaca 4 library, the SGMT clade and RO clade sequences constituted 74.9% and 17.9% of clones, while in the alpaca 8 library, the SGMT and RO clades showed a 59.8% and 31.7% representation, respectively (Figure 3). In light of this observation, we re-examined previously published data by our group to compare the sequence distribution between the SGMT clade and the RO clade from other host species. We have found that the SGMT clade is more dominant than the RO clade in sheep from Venezuela (SGMT: 62.5%; RO: 32.7%) [28] and in reindeer (SGMT: 44.8%; RO: 2.3%) [5]. In strong contrast, the RO clade is distinctively more highly represented than the SGMT clade in the hoatzin (SGMT: 0%; RO: 85.8%) [6], and in corn-fed cattle from Ontario (SGMT: 4%; RO: 48%) [31]. In light of these observations, *Methanobrevibacter *phylotypes which are highly dominant in sheep from Venezuela and in the hoatzin for instance, accounting respectively for 95.2% and 85.8% of the methanogens identified in these hosts, are in fact very dissimilar when we analyze the distribution of phylotypes between the SGMT and RO clades.

## Conclusions

While additional studies are required to elucidate the respective contributions of host species genetics and environmental factors in the determination of whether the SGMT or the RO clade will be the most highly represented in a microbial population, they may represent methanogen groups that thrive in different conditions. For instance, factors such as rumen or forestomach pH, tolerance to toxic compounds, and the rate of passage can act as selection agents, either individually or in combination, by promoting the growth of particular groups of methanogens, thereby affecting the population structure of the archaeal community [38]. From the available rumen methanogen 16S rRNA gene public dataset, Kim et al. [3] conservatively identified 950 species-level OTUs, and it has been predicted that many novel archaea still remain to be identified. In this context, the natural division of *Methanobrevibacter*-like sequences into the SGMT and RO clades could prove useful in developing population structure models for foregut methanogens that take into account phylogeny and representation. Improved population models could then be tested for methane production under controlled conditions *in vivo *or *in vitro*. This strategy may therefore prove to be very valuable in the design of broad range mitigation strategies in the future.

## Authors' contributions

BS performed DNA extractions, PCR amplification of methanogen 16S rRNA genes, clone library construction, data analysis, and drafted the manuscript. ADW conceived the study, sampled forestomach contents from animals, performed data analysis and drafted the manuscript. All authors read and approved the final manuscript.

## Supplementary Material

Additional file 1 **Table S1**. List of individual 16S rRNA gene sequences identified in the forestomach of the alpaca and their corresponding GenBank accession. Identical sequences found more than once are indicated and grouped under a single representative with the same accession. Click here for file
